# Case Report: A Novel In-Frame Deletion of *GLIS2* Leading to Nephronophthisis and Early Onset Kidney Failure

**DOI:** 10.3389/fgene.2021.791495

**Published:** 2021-11-30

**Authors:** Intisar Al Alawi, Laura Powell, Sarah J. Rice, Mohammed S. Al Riyami, Marwa Al-Riyami, Issa Al Salmi, John A. Sayer

**Affiliations:** ^1^ Translational and Clinical Research Institute, Faculty of Medical Sciences, Newcastle University, Newcastle upon Tyne, United Kingdom; ^2^ National Genetic Center, Ministry of Health, Muscat, Oman; ^3^ Biosciences Institute, Faculty of Medical Sciences, Newcastle University, Newcastle upon Tyne, United Kingdom; ^4^ Pediatric Nephrology Unit, Department of Child Health, Royal Hospital, Ministry of Health, Muscat, Oman; ^5^ Department of Pathology, College of Medicine, Sultan Qaboos University, Muscat, Oman; ^6^ Renal Medicine Department, Royal Hospital, Ministry of Health, Muscat, Oman; ^7^ Internal Medicine, Oman Medical Specialty Board, Muscat, Oman; ^8^ Renal Services, Newcastle Upon Tyne Hospitals NHS Foundation Trust, Newcastle upon Tyne, United Kingdom; ^9^ Newcastle Biomedical Research Centre, NIHR, Newcastle upon Tyne, United Kingdom

**Keywords:** nephronophthisis (NPHP), whole exome sequencing, end stage kidney disease (ESKD), consanguinity, ciliopathies

## Abstract

Variants in the *GLIS family zinc finger protein 2* (*GLIS2*) are a rare cause of nephronophthisis-related ciliopathies (NPHP-RC). A reduction in urinary concentration and a progressive chronic tubulointerstitial nephropathy with corticomedullary cysts are the major characteristic features of NPHP. NPHP demonstrates phenotypic and genetic heterogeneity with at least 25 different recessive genes associated with the disease. We report a female, from a consanguineous family, who presented age 9 years with echogenic kidneys with loss of cortico-medullary differentiation and progressive chronic kidney disease reaching kidney failure by 10 years of age. A novel homozygous in-frame deletion (NM_032,575.3: c.560_574delACCATGTCAACGATT, p.H188_Y192del) in *GLIS2* was identified using whole exome sequencing (WES) that segregated from each parent. The five amino acid deletion disrupts the alpha-helix of GLIS2 zinc-finger motif with predicted misfolding of the protein leading to its predicted pathogenicity. This study broadens the variant spectrum of *GLIS2* variants leading to NPHP-RC. WES is a suitable molecular tool for children with kidney failure suggestive of NPHP-RC and should be part of routine diagnostics in kidney failure of unknown cause, especially in consanguineous families.

## Introduction

Nephronophthisis (NPHP) is an autosomal recessive inherited kidney disease that constitutes the most prevalent monogenic causes of kidney failure in the first 3 decades of life and is responsible for 2.4–15% of pediatric patients with kidney failure (Hildebrandt et al., 2009; Luo and Tao 2018). Urine analysis of NPHP patients generally does not illustrate any characteristic urinary abnormalities, as proteinuria and hematuria are often not found until late stages of chronic kidney disease (CKD) (Luo and Tao 2018). A clue to the underlying diagnosis may be finding a loss of urinary concentration ability (Simms et al., 2009) and kidney ultrasound scan findings of corticomedullary cysts and a loss of corticomedullary differentiation (Srivastava et al., 2017); however, these findings are non-specific.

Therefore, it is difficult to detect NPHP using simple laboratory testing and often the diagnosis is delayed until the advanced stages of CKD. A kidney biopsy may show features which include tubular atrophy, tubular basement defects, and interstitial fibrosis but often do not lead to a precise diagnosis, especially if the patient is approaching kidney failure (Simms et al., 2009). Extra-renal manifestations occur in 15% of cases of NPHP, consistent with it being a ciliopathy syndrome and may include retinal defects, liver fibrosis, skeletal defects, and brain developmental disorders (Srivastava et al., 2017).

Recent advances in genetic analysis show excessive phenotypic and genetic heterogeneity of this disorder (Chaki et al., 2011; Tang et al., 2020) with at least 25 different genes having been linked (Luo and Tao 2018; McConnachie et al., 2021). However, pathogenic variants in known NPHP genes can explain only up to one-third of cases of clinically diagnosed nephronophthisis, suggesting further genes may underlie this phenotype (König et al., 2017; Luo and Tao 2018). Here, we report a case of NPHP associated with early kidney failure caused by a homozygous in-frame deletion of *GLIS2* diagnosed using WES. Pathogenic variants in *GLIS2* remain very rare with only two families previously reported (Attanasio et al., 2007; Halbritter et al., 2013). This case is unique, as it is the first in-frame deletion within *GLIS2* to be reported in association with NPHP and the predicted loss of a motif within the zinc finger domain suggests strongly its pathogenicity.

### Case Description

**FIGURE 1:**
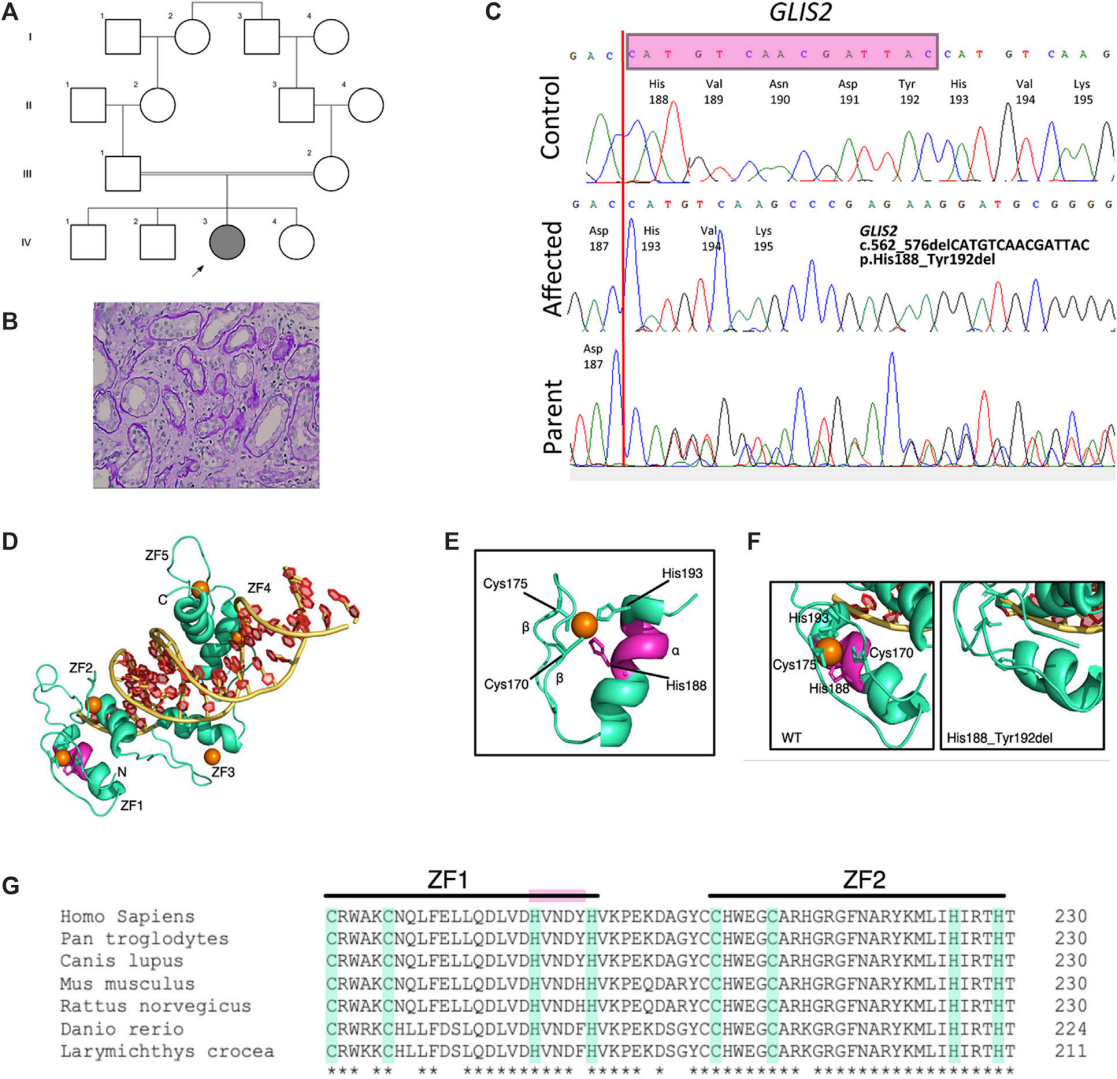
Identification and in silico analysis of novel *GLIS2* variant leading to nephronophthisis. **(A)** Pedigree diagram showing proband (arrowed) and known family consanguinity. **(B)** Kidney biopsy histology from proband showing dilated tubules and thickening and duplication of tubular basement membranes (hematoxylin and eosin, x40). **(C)** Sequence chromatograms demonstrating homozygous in-frame deletion in *GLIS2* (NM 032575, c.562_576delCATGTCAACGATTAC; p.His188_Tyr192del) which was heterozygous in both parents. **(D–G)**. In-frame deletion of *GLIS2* is predicted to destroy the terminal motif sequence within the first zinc finger. **(D)** Protein model of human GLIS2 (green) bound to DNA (yellow and red) using structural homology to human GLI protein (PDB accession 2GLI). The predicted binding positions of Zn ions to each of the five zinc-finger (ZF) domains (labelled ZF1-5) are indicated by orange circles. **(E)** The predicted structure of GLIS2 ZF1 showing the classical ββα fold. The C_2_H_2_ residues Cys170, Cys175, His188, and His193 are labelled, and side chains are displayed. The five amino acid residues deleted in the His188_Tyr192 deletion are highlighted in magenta. **(F)** Zoomed in images from **(D)** of ZF1, showing the predicted wild-type structure **(left)** and predicted structure in the mutated protein **(right)**. Left, the C_2_H_2_ residues are labelled and side chains creating the zinc binding pocket are shown. The region deleted in the mutated protein is highlighted in magenta. Right, His193 replaces His188 in the zinc binding pocket, however, the classical ZF motif is lost in the His199_Tyr192del variant. **(G)** Multiple sequence alignment of GLIS2. The C_2_H_2_ motifs of ZF1 and ZF2 are highlighted in green. The deleted sequence in the mutated protein is highlighted in magenta. *, completely conserved residue.

The proband initially presented at 9 years of age to an orthopedic surgeon following a right hip injury. At this stage she was noted to be hypertensive and had evidence of kidney failure, chronic kidney disease (CKD) stage 4 (Table 1). Urine analysis was bland. She was commenced on amlodipine and oral sodium bicarbonate to correct a metabolic acidosis. An ophthalmology assessment revealed no evidence of uveitis or retinitis pigmentosa, and hearing was normal. Parental consanguinity was reported (Figure 1A).

**TABLE 1:** Timeline of patient’s journey.

	Presentation	Progression	Progression
**Age**	9 years old	10 years old	11 years old
**CKD stage**	CKD stage 4	CKD stage 5	CKD stage 5, commenced hemodialysis
**eGFR** (ml/min/1.73m^2^)	24	15	12
**Investigations**	Bland urine analysis	Renal biopsy showing NPHP	Molecular genetic studies

CKD, chronic kidney disease; eGFR, estimated glomerular filtration rate (Revised Schwartz equation); NPHP, nephronophthisis.

Kidney ultrasound scanning showed normal sized but echogenic kidneys with loss of cortico-medullary differentiation. A 24-h urine collection did not show significant proteinuria. To intimately investigate the cause of progressive kidney failure, a kidney biopsy was performed which demonstrated tubular injury, tubulointerstitial nephritis, interstitial fibrosis, and tubular atrophy suggesting NPHP (Figure 1B). Immunofluorescence studies were negative. Kidney function deteriorated and she reached ESRD by the age of 10 years. We proceeded to investigate the family, following informed consent for genetic studies.

## Materials and Methods

### Patients

The affected patient, her parents and the eldest of the unaffected siblings were recruited from an Omani cohort of patients within the Royal Hospital, Ministry of Health Hospital, Muscat, Oman with suspected inherited renal ciliopathy syndrome. This study was approved by the North-East Newcastle and North Tyneside 1 Research Ethics Committee (18/NE/350). Whole blood (1.5–2.5 ml in EDTA) samples were collected specifically for this study and used for extraction of genomic DNA. DNA samples from affected and other family members were given a pseudo-anonymized sample number. Written and informed consent was obtained from the parents/guardians of each patient, and any family members (including parents and siblings) involved in this study.

### Genetic Studies

Genetic analysis using whole exome sequencing (WES) was carried out after obtaining consent from the family. Whole-exome sequencing was performed in the proband and was outsourced to Novogene Co., Ltd. (China) as previously described (Al Alawi et al., 2021). Exomes were captured by SureSelect Human All Exon V6 Enrichment Kit (Agilent Technologies, CA, United States), and high-throughput sequencing was conducted with an Illumina HiSeq platform (Illumina, San Diego, CA). Analyses of raw data (FASTQ format) were achieved together with sequence reads mapping to the human reference genome hg19 using BWA (Li and Durbin 2009), removal of PCR duplicates using Picard (http://broadinstitute.github.io/picard/), alignment refinement using GATK, and coverage analysis and SNP and INDEL calling using GATK’s Haplotype Caller (McKenna et al., 2010). SNP and INDEL VCF files were examined using Qiagen QCI Interpret Translational tool for variants filtration and annotation. The approach of data filtering was as follows: (1) non-synonymous SNPs and frameshift/in-frame INDELs with an alternative allele frequency >0.01 in the NHLBI Exome Sequencing Project, Single Nucleotide Polymorphism database (dbSNP), 1000 Genomes Project (1000G), the Genome Aggregation Database (gnomAD) were excluded; (2) filtered SNVs and INDELs, predicted by in-silico algorithms to be damaging, were maintained; (3) variants classified as pathogenic and likely pathogenic according to American College of Medical Genetics (ACMG) guidelines remained (Richards, Aziz et al., 2015); and (4) co-segregation analysis was performed in the family using Sanger sequencing. Genetic variants were submitted to ClinVar (www.ncbi.nlm.nih.gov/clinvar).

## Results

Whole exome sequencing of the proband’s genomic DNA was performed with 96.4% of the exome being covered at least 20-fold. Variants were filtered and analyzed as described, followed by Sanger sequencing validation. Within a large region of homozygosity on chromosome 16, a homozygous novel in-frame deletion variant (NM_032,575.3:c.560_574delACCATGTCAACGATT, p.H188_Y192del) in *GLIS2* was identified in the patient and was present in its heterozygous form in both parents but absent in the eldest unaffected sibling from whom DNA was available (Figure 1C). We did not find any other potential pathogenic variants associated with a ciliopathy-related phenotype. Variants in *GLIS2* have been previously identified in patients with NPHP (OMIM: 611498) who developed kidney failure in early childhood (Attanasio et al., 2007). This novel deletion variant that leads to a shortened protein missing 5 amino acids (H188, V189, N190, D191, Y192) located in exon 4 of the *GLIS2* gene was absent in the public database including 1000G and genomAD. Bioinformatics programs including PROVEAN (deleterious), SIFT (deleterious), and MutPredIndel (score: 0.45615) predicted that this variant (NM_032,575.3:c.560_574delACCATGTCAACGATT, p.H188_Y192del) is deleterious and located at evolutionarily conserved site of the GLIS2 protein close to the zinc-finger C2H2-type DNA-binding domain (Figure 1D). According to ACMG guidelines (Richards et al., 2015), this allele is classified as a variant of uncertain significance (VUS). The variant has been submitted to ClinVar (https://www.ncbi.nlm.nih.gov/clinvar/variation/1299703/).

Gli-similar 2 (GLIS2) is a member of the Krüppel-like family of transcription factors consisting of five contiguous zinc-finger motifs (Figure 1D) (Zhang et al., 2002). Classical zinc-finger domains (C_2_H_2_) are among the most common eukaryotic protein domains and often have the consensus sequence Cys-X_2–4_-Cys-X_12_-His-X_2–6_-His (where X is any amino acid) (Pavletich and Pabo 1993). Metalloproteins, such as GLIS2 have been shown to co-assemble with Zn^2+^ ions during protein folding into the ββα zinc finger domains (Figure 1E). The His188_Tyr192 variant identified in this study removes 5 amino acids from the *α* helix of GLIS2 ZF1 (Figure 1D, magenta), removing the terminal sequence of the zinc-finger motif, even though His193 replaces His188 as H_1_ (Figures 1E–G). The loss of this motif through an in-frame deletion could potentially result in misfolding of GLIS2. However, there is evidence to show that the individual zinc-finger domains fold independently of the bulk protein structure (Cox and McLendon 2000), and therefore an observed loss of function could be due to altered DNA-binding capacity of the *GLIS2* mutant. Downstream functional analyses are required to determine the loss-of-function mechanism.

## Discussion

Making a clinical diagnosis of NPHP is difficult given its non-specific features and therefore many patients with NPHP may be misdiagnosed with another nephropathy or diagnosed as CKD of unknown etiology (Srivastava and Sayer 2014; Snoek et al., 2018). Although clinical findings, kidney ultrasound examination, and kidney biopsy histology in our patient were suggestive of NPHP, a definitive diagnosis was only made following molecular genetic testing. In fact, as many causes of kidney failure may mimic the NPHP phenotype, molecular genetic testing is critical to distinguish NPHP from other inherited forms of kidney diseases.

NPHP is a rare disorder (Levy and Feingold 2000; Hildebrandt 2010) that illustrates genetic heterogeneity with at least 25 different recessive genes having been linked with the disease (Luo and Tao 2018). Almost all NPHP genes encode nephrocystins that localize to primary cilia, except for NPHP-like genes such as *XPNPEP3* and *SLC41A1* and *GLIS2* genes, with protein products located to mitochondria and nucleus, respectively (O'Toole et al., 2010; Srivastava and Sayer 2014; Luo and Tao 2018). Pathogenic variants in these genes can explain only up to one-third of cases and around 60% of cases remain genetically unsolved, suggesting additional genetic causes that have yet to be revealed (König et al., 2017; Luo and Tao 2018). The most common genetic cause of NPHP is variants in *NPHP1*, associated with 20% of cases (Hildebrandt et al., 2009), while variants in each of the remaining genes possibly contribute up to 1% of cases (Srivastava and Sayer 2014; Luo and Tao 2018).

The human *GLIS2*, encoding a member of the Kruppel-like zinc-finger protein family named GLIS family zinc-finger two or alternatively nephrocystin-7*,* is located on chromosome 16p13.3, and it contains 6 exons spanning ∼3.7 kilobases. GLIS2 is located in the nucleus and cytoplasm and is mainly expressed in the kidney and weakly in the heart, lung, placenta, and colon (Zhang et al., 2002). Based on the cell context, GLIS2 can function as activator or repressor of gene transcription. For instance, it works as a repressor of the Hedgehog (Hh) signaling pathway by repressing Hh-dependent expression of Wnt4 as well as repressing transcriptional activation mediated by CTNNB1 in the Wnt signaling pathway. In addition, GLIS2 is required to retain the differentiated epithelial phenotype in kidney cells and in neuron differentiation (Zhang et al., 2002).

In 2007, a homozygous splice-site variant in *GLIS2* (c.755+1G > T) was identified in three affected Canadian Oji-Cree children with an autosomal recessive pattern of NPHP (Attanasio et al., 2007). Later, in 2013, a homozygous missense *GLIS2* variant (c.523T > C, p.C175R) was identified in a Turkish patient with isolated NPHP who reached kidney failure aged 15 years, as part of a worldwide cohort screen of patients with NPHP-RC (Halbritter et al., 2013). Variants in *GLIS2* have been classified as NPHP7 (OMIM 611498) and within the variation database of ClinVar, there are 34 pathogenic variants, of which 31 are copy number variants (CNVs) while three are SNVs (two missense and one splicing) and there are 92 variants of uncertain significance (VUS). Here, we identified in *GLIS2* an in-frame deletion variant (NM_032,575.3: c.560_574delACCATGTCAACGATT, p.H188_Y192del) in Omani family with NPHP and early onset kidney failure. As far as we know, this variant is novel and expands the variant spectrum of *GLIS2* variants associated with NPHP.

The deletion affects the zinc-finger C2H2-type DNA-binding domain of GLIS2, which plays a role in the recognizing and binding to target DNA as well as mRNA and protein targets (Brayer and Segal 2008). This deletion leads to a change in protein length and feature as the position of the stop-codon in mutated protein will be 520 compared to 525 in the wild-type protein. Further functional studies may be performed in the future to examine the consequences of such deletion in *GLIS2* expression level.

In summary, by employing WES, we detected a novel in-frame deletion (NM_032,575.3: c.560_574delACCATGTCAACGATT, p.H188_Y192del) in *GLIS2* in an Omani family with NPHP and kidney failure. Therefore, this study has not only broadened the spectrum of variants within *GLIS2*, but also extended the molecular pathogenesis spectrum of kidney disease in Oman (Al Alawi et al., 2021) and allowed the confirmation of the clinical diagnosis of NPHP leading to kidney failure.

## Data Availability

The original contributions presented in the study are publicly available. This data can be found here: GenBank accession number: BankIt2508176 GLIS2 OK482597.
